# Stakeholder views on a recovery-oriented psychiatric rehabilitation art therapy program in a rural Australian mental health service: a qualitative description

**DOI:** 10.1186/s13033-015-0005-y

**Published:** 2015-03-10

**Authors:** Nadia De Vecchi, Amanda Kenny, Susan Kidd

**Affiliations:** La Trobe Rural Health School, La Trobe University, Bendigo, VIC 3552 Australia; Psychiatric Services Professional Development Unit, Bendigo Health, Bendigo, VIC 3552 Australia

**Keywords:** Art therapist, Art therapy, Consumers, Mental health services, Nurse managers, Recovery, Rehabilitation

## Abstract

**Background:**

Recovery-oriented care is a guiding principle for mental health services in Australia, and internationally. Recovery-oriented psychiatric rehabilitation supports people experiencing mental illness to pursue a meaningful life. In Australia, people with unremitting mental illness and psychosocial disability are often detained for months or years in secure extended care facilities. Psychiatric services have struggled to provide rehabilitation options for residents of these facilities. Researchers have argued that art participation can support recovery in inpatient populations. This study addressed the research question: Is there a role for the creative arts in the delivery of recovery-oriented psychiatric rehabilitation for people with enduring mental illness and significant psychosocial disability detained in a secure extended care unit?

**Methods:**

The study had two major aims: to explore the experiences of consumers detained in a rural Australian secure extended care unit of an art therapy project, and to examine the views of nurse managers and an art therapist on recovery-oriented rehabilitation programs with regard to the art therapy project. A qualitative descriptive design guided the study, and a thematic network approach guided data analysis. Ethics approval was granted from the local ethics committee (AU/1/9E5D07). Data were collected from three stakeholders groups. Five consumers participated in a focus group; six managers and the art therapist from the project participated in individual interviews.

**Results:**

The findings indicate that consumer participants benefitted from art participation and wanted more access to rehabilitation-focussed programs. Consumer participants identified that art making provided a forum for sharing, self-expression, and relationships that built confidence, absent in the regular rehabilitation program. Nurse manager and the art therapist participants agreed that art participation was a recovery-oriented rehabilitation tool, however, systemic barriers thwarted its provision.

**Conclusions:**

The transformation of mental health services towards a recovery orientation requires commitment from service leaders to provide evidence-based programs. Psychiatric rehabilitation programs based on local need should be included in public mental health services. This study supports the use of art-based rehabilitation programs for people detained in rural secure extended care facilities. Introducing these programs into clinical practice settings can improve the consumer experience and support organisational culture change towards a recovery orientation.

## Background

Recovery-oriented service provision is a direction for mental health services across Western nations and is mandated throughout Australia. At organisational and individual levels, recovery-oriented service provision enhances recovery processes for people diagnosed with enduring mental illness [1]. Psychiatric rehabilitation incorporates principles of recovery to support people to pursue meaningful lives [2]. Art-based rehabilitation programs support the recovery journeys of people with enduring mental health issues in inpatient and community settings [3]. In Australia, recovery is defined as “being able to create and live a meaningful and contributing life in a community of choice with or without the presence of mental health issues” [1].

In Victoria, Australia, care provision for a small percentage of people with enduring psychiatric illness and psychosocial disability is on an involuntary basis in secure extended care units (SECU). Involuntary residency in a SECU can span months and years, despite assessment, treatment, and recovery-oriented rehabilitation programs [4]. Data on SECU recovery-oriented rehabilitation outcomes are sparse. Consumers of rural mental health services are particularly disadvantaged by inconsistent access to evidence-based programs, distance to programs, poorer health, and social isolation [4,5]. This article addresses the research question: Is there a role for the creative arts in the delivery of recovery-oriented psychiatric rehabilitation for people with enduring mental health issues and significant psychosocial disability detained in a secure extended care facility? The experiences of consumers, and the views of nurse managers and an art therapist on the “Making Precious Things” project, a recovery-oriented psychiatric rehabilitation art therapy program delivered in a rural Australian SECU, are described to address the research question.

### Recovery

Varied notions of recovery exist across stakeholder groups in mental health services [6]. “Recovery in” [7], locates consumer/survivor/ex-patient definitions within a social model of disability, and preferences personal recovery and the pursuit of human rights and citizenship. Survivors of mental health services consider themselves experts on their recovery needs and position themselves at the centre of their treatment, exercising their right to personal choice, self-determination, and participation. “Recovery from” [7], focuses on biomedical classifications, treating disease, eliminating symptoms, and reducing medical disability. With treatment frameworks for delivering paternalistic interventions if the disease process is so disabling that the person is an unable to live independently in the community [6]. Focussed on individual pathology, cure and remission, recovery from is not concerned with addressing broader social agendas of social justice and stigma [8].

### Recovery-oriented psychiatric rehabilitation

Recovery is a personal journey of hope, empowerment, connection with peers and the community, developing an identity beyond illness, and living a meaningful life [9]. Recovery-oriented psychiatric rehabilitation offers a long-term model for people to support their pursuit of meaningful community roles, combining treatment and rehabilitation in “an active process of self-agency” [8]. Recovery-oriented rehabilitation practice requires clinicians to develop working relationships that place consumers at the centre of care [10], with clinicians using interventions to support the identification of strengths and self-determined goals [2]. Evidence-based interventions include supported housing, employment and education, peer support, residential and group programs, including therapeutic modalities [8] with art therapy becoming recognised [11].

### Art participation in mental health recovery

Qualitative research indicates that creative art programs can support recovery in people with enduring mental health issues [3,12]. A study in a psychiatric inpatient setting in the United Kingdom (GB) interviewed 11 consumers, and using a narrative analysis approach, reported that art participation provided people with a therapeutic and creative social environment, and improved confidence, a sense of self, and hope [12]. Another study that interviewed 35 participants from six art participation projects across GB, as part of a larger case study examining the use of art participation in mental health recovery, concluded that art participation contributed to combatting stigma, provided peer support, and opportunities for an identity beyond that of mental illness [13].

In a critical analysis of the mental health literature spanning 1987 to 2011, van Lith et al. identified a range of benefits stemming from art participation across clinical, personal, functional, social, occupational, and environmental dimensions of recovery [3]. However, randomised controlled trials (RCT) investigating the benefits of art therapy for people living with schizophrenia have found minimal improvements [14,15]. A Cochrane review concluded that establishing benefits in this population required more research [16]. Despite this gap in evidence, guidelines for the treatment of schizophrenia recommend the use of art therapy in clinical settings [17].

### Challenges of providing recovery-oriented services

Recovery-oriented service provision is difficult in SECU type facilities that are predominately focussed on risk management, protecting the community, and containment [18,19]. Many scholars propose transformation in mental health organisations for recovery to become embedded in service provision structures and practices [20,21]. According to Pilgrim, coordinated consultation of all stakeholders in a local area can determine their epistemological positions allowing examination of the issues that prevent recovery-oriented practice [6]. Identifying, articulating, and making space for dialogue about these tensions supports the development of recovery-oriented approaches and contributes to transformation in mental health services [22]. Understanding the way that a local service makes sense of their epistemological framework underpins the change process [6].

Whilst consumer participation is mandated in all areas of service provision under policy guided by recovery-oriented principles [1,23], services have been slow to demonstrate their uptake of this process [24] and there is a dearth of literature that examines the experiences of people detained in secure extended care facilities [4]. This study is unique because it captures the experience of consumers living with enduring mental health issues and significant psychosocial disability detained in a SECU. The views of an art therapist were seen as important to provide insights into the use of art making in recovery-oriented psychiatric rehabilitation. Their perspective has been captured in only a small number of studies [25].

The views of nurse managers on recovery-oriented psychiatric rehabilitation programs were deemed important given their leadership role in local organisational culture and policy development [26], which is particularly relevant for consumers detained in secure extended care. However, there is a lack of research that explores their views on recovery-oriented rehabilitation [27]. The ability of service managers to articulate their vision for recovery-oriented rehabilitation programs can provide the impetus toward evaluating current processes, and where necessary, implementing change processes [1]. Whilst the Making Precious Things project was conducted in one service, the usefulness of evaluating local recovery-oriented projects for broader learning has been identified [28-30].

### The making precious things project

The Making Precious Things project was conducted over 20, one hour weekly sessions between January and May 2012. Different visual art forms and media were explored, focussing on the process of creating art. Two art therapists were employed, and the student researcher (ND) attended as an observer and participant. Consumer attendance was voluntary, with five to eight participants (20-40% of unit population), and a core group of three long-term residents. The group had a recovery-oriented rehabilitation focus aimed at creating a safe and respectful space for participants to share stories, make art, and feel valued. Each session involved an introduction to the topic and medium, with art therapists offering therapeutic guidance on subjects that enabled the exploration of identity. The concluding minutes of each session were devoted to group reflection, where personal meaning was drawn from the artworks. A group exhibition celebrated the end of the project, and group members shared their participation through selected artworks with family, carers, and staff.

## Methods

### Aim

This study had two major aims. Firstly, to explore the experiences of consumers who participated in the Making Precious Things project, a recovery-oriented psychiatric rehabilitation art therapy program, delivered in a secure extended care facility of a rural Australian public mental health service. Secondly, to examine the views of nurse managers and an art therapist on recovery-oriented rehabilitation programs in the context of the Making Precious Things project.

### Design

Qualitative descriptive design aims to provide a straight, rich description of participants’ experiences of real-world events. The approach aims to provide close to the surface meaning of the collected data, with a minimal degree of data interpretation. Qualitative descriptive studies have significance for clinicians and policy makers because they can be used to inform future interventions, practice, and research projects using the multifaceted understanding of an experience [31].

### Participants

Consumer participants were recruited using purposive sampling during the concluding exhibition to provide a lived experience perspective of the art therapy program. At the commencement of the celebratory showing of the project’s art works, a focus group was advertised, and participant information and consent forms were distributed. One researcher (AK) explained participation and consent processes, stipulating the voluntary nature. Staff and carers supported consumers to understand their decision to participate before written consent was obtained. The focus group was held after the celebration with five consumer participants electing to participate.

Purposive maximum variation sampling was used to recruit nurse managers within the organisation. This provided a sample rich in information [31] on managers’ experience of the provision of art-based recovery-oriented psychiatric rehabilitation. Managers were deemed to be an important group to include, as they influence policy governing the programs on offer and their leadership is important in driving organisational transformation towards a recovery orientation. The Executive Director of the service invited managers to participate, and potential participants contacted the researchers, to complete consent processes prior to the interview. The lead art therapist from the Making Precious Things program was recruited by invitation because her insider role in art therapy and her outsider position to the organisation provided a different perspective that was important to capture. Recruitment and interviewing of managers continued until the research team deemed no new information was forthcoming after careful reading and re-reading of the transcripts, and data saturation had been achieved.

### Data collection

Data were collected between July 2012 and January 2013 via two semi-structured interview guides. Consumers participated in a one-hour focus group because they were accustomed to working in a group. Questions for the focus group included: which parts of the creative art program did you enjoy/not enjoy? Why? Do you attend any other rehabilitation programs? In what way is the creative art program different/the same? Do you think creative art programs assist in your rehabilitation? What prevents you from participating in programs? What group programs would you like to attend? An experienced researcher (AK) conducted the focus group, with two other researchers present to support the process. The presence of three researchers was considered appropriate ethically. The participants knew two of the researchers, ND via a direct care relationship, and SK in an educator role within the facility. The senior researcher was not employed by the service nor known to participants.

Individual interviews with nurse managers and the art therapist were conducted by two of the researchers (ND, SK). Interviews were between 40 to 60 minutes. Interview questions included: describe your experience of the role and availability of art-based psychiatric rehabilitation in this clinical mental health service. Do you see benefits from providing art-based rehabilitation programs in clinical services? What are the barriers/facilitators in offering creative art rehabilitation programs in this service? All interview sessions were audio recorded and transcribed and returned to interviewees for confirmation of accuracy. No changes were made to original transcripts.

### Analysis

**Figure 1:**
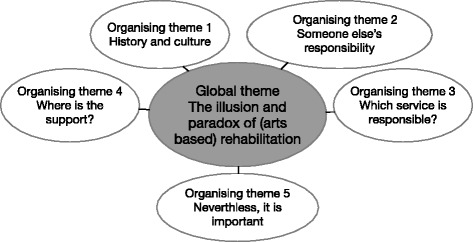
**Thematic network global theme development.** Themes branch inwards towards the central global theme in a non-hierarchical web of metaphors derived from the data.

Thematic network analysis guided the interpretation of the data [32]. Analysis involved grouping increasingly abstract interpretations of data using an iterative process of careful reading and rereading of the text to identify repeated ideas and issues related to the study aim. Initial basic themes were grouped and regrouped under broader inclusive organising themes, providing the basis for the super-ordinate global theme that described the overall metaphors contained in the data groupings extracted. The developed networks were explored and described in a logical, narrative sequence, through the lens of the global, organising and basic themes, using text segments to illustrate and support the analysis. Figure 1: Thematic network global theme development, describes the relationship between the data analysis groupings for the managers and art therapist interviews. Two authors (ND, SK) undertook the analysis independently; initial immersion was recorded in a table, with successive immersions represented in a poster format with sticky notes. The team deliberated over the interpretations until agreement was achieved. No significant differences were found in the data groups.

### Trustworthiness

Trustworthiness was addressed using Lincoln and Guba’s criteria [33]. Trustworthiness was demonstrated by a team approach to data collection, analysis and data saturation, and researcher reflexivity evidenced by a research audit trail, supervision meetings, and retained email correspondence between the research team. Member checking, a thorough literature review, immersion in the data during analysis, sampling of data-rich participants, use of a focus group, flexible interview style, accurate transcription, and a structured analysis that remained close to the data were all important.

### Ethics and ethics approval

The major ethical consideration was working with a vulnerable group. The following considerations were made: a focus group format held in the same room as the art sessions because consumer participants were familiar with the group process, each other, two of the researchers (ND, SK), and the environment. Confidentiality of participants is protected using pseudonyms. Ethics approval was obtained from the health service Human Research Ethics Committee and the Victorian Department of Health (AU/1/9E5D07).

## Results

### Consumer focus group

All participants had attended six or more art sessions with the gender mix, cultural identity, age range, and diagnosis representative of the unit population. Four males and one female participated, including the three core art therapy group members. Participants were aged between 21 and 45 years, all Anglo-Saxon in origin and residents of surrounding rural communities. All participants had a diagnosis of a psychotic disorder, and comorbidity of drug and alcohol misuse, acquired brain injury, or intellectual disability. The global theme *relational practice creates community* comprised four organising themes: *doing art therapy*, *personal reflections*, *groups in an individual environment*, and *generating connection*. Participants were enthusiastic about access to inpatient art participation and recovery-oriented rehabilitation activities.

#### Doing art therapy

This theme explores the notion that participation in creative art programs has potential benefit for the personal recovery of consumers with enduring mental health issues when detained in services. Consumer participants described art therapy as enjoyable, because it provided opportunities to work together creatively. Discussion, choice, negotiation and compromise, were recognised as essential to plan, develop, and complete artworks. All but one participant described an improved appreciation of others’ perspectives and working together. Most thought that making artwork together encouraged sharing ideas, thinking creatively and learning from and about each other. Participants identified that the creative endeavour had developed a sense of community. Participant 2 elaborates:

*I think it was in a group we*’*re all different*, *and we have illnesses in the mental. I think we are all different. What I am trying to say is we are all equal*.

Several participants described feeling accepted, which supported their creative expression. Doing and sharing artwork was experienced as encouraging creative exploration of personal meanings, emotions, and concepts of self. Participant 1 explains:

*No*, *the art*’*s kind of*, *compared to talking it wouldn*’*t be the same* ‘*cos everyone can do art really*, *everyone can have a go and that sort of thing*, *and sitting and talking and having a discussion tends to be a lot harder work than doing a piece of art yourself. The art gives you a way to express yourself rather than just talking about yourself to people in a group*.

All participants said they felt comfortable in the group setting. They identified that the congenial atmosphere of the group supported peoples’ self-confidence, because there was no pressure to perform, and they received immediate feedback on their work. Participants described feeling more confident in life as a result of their participation, the art space provided a place where they could be distracted from worries, and where completing an art piece was possible. This experience of accomplishment and success motivated participants to return to the group. Participants expressed pride in exhibiting their artwork, and several related that the art making process had enabled learning that life offered possibilities for positive risk taking and the discovery of personal interests. Participant 1 identified guidance as instrumental:

*Having a project makes you feel good about the group really* – *it*’*s better than being given a piece of paper and told to draw something*, *there wouldn*’*t be much going on really. But having a project set out before you start sort of makes the whole group*.

#### Personal reflections

This theme explores the notion that personal challenges experienced by consumers with enduring mental health issues impede their participation in creative art programs as part of their recovery-oriented rehabilitation. Participants reflected on personal barriers of reluctance to becoming involved, fear and withdrawal in attending group programs. They identified that amotivation and low self-esteem hindered participation, and participant 3 believed support was necessary. All but one felt that working with familiar people in an inpatient setting offered mutual support with less fear.

#### Groups in an individual environment

This theme reflects the notion that barriers exist within the service and culture that prevent the provision of recovery-oriented rehabilitation programs in psychiatric facilities. Participants described systemic barriers to art-based and other rehabilitative group work. Forming a therapeutic and inclusive art therapy group was experienced, by all participants, as different from standard unit care and culture because of the opportunities to experiment, learn, and experience motivation. Participant 2 explains:

*I think it was different experiencing different ideas and pieces of artwork*, *that you and other people around you experienced*, *and their ideas and getting it on paper or with a picture*, *just the different ideas of everyone else and your own*.

All participants appreciated the therapeutic nature and community spirit of the art therapy group. Participants identified a lack of opportunities for group work in the service generally. Participant 1 elaborates:

*If there were computers [here] I could run a group myself on using computers. I have done that before in a previous job a while ago*…*It would be really rewarding*, *you could help people out*, *and watch people learn and grow and develop skills that you had taught*, *and they could get enthusiastic about it*, *and that would be good*.

#### Generating community

This theme outlines the notion that while consumers experience enduring mental health issues they want choices in care that includes participating in recovery-oriented group rehabilitation programs while detained in inpatient units. Participants suggested improvements to art therapy including: different materials and methods, longer sessions with more complicated projects, and making art in nature. They also made suggestions for other group work they would like included in the service: making computer dance music; bushwalking and adventure; cooking and sports. Participants described wanting support with getting out, socialising, exploring self and others, improving their self-confidence, and in finding work.

### Managers and the art therapist interviews

Participants comprised one art therapist and six nurse managers. The nurse managers were all male and included middle managers of inpatient and community teams and high-level managers in education and directorship. The art therapist was invited from the Making Precious Things project and had seventeen years experience working with people with long-term mental health issues in Australia and Europe. The analysis produced the global theme: *the illusion and paradox of* (*art*-*based*) *rehabilitation*. Five organising themes, built the global theme’s foundation: *history and culture*, *someone else*’*s responsibility*, *which service is responsible*?, *where is the support*?, and *nevertheless*, *it is important*. The findings indicate that systemic barriers hinder the provision of art-based recovery-oriented rehabilitation programs, despite identified benefits to people with enduring mental illness.

#### History and culture

This theme reflects the unacknowledged notion that the history of psychiatry continues to negatively impact the culture of mental health services particularly for people with enduring mental health issues detained in the service. Participants described their experience of all forms of inpatient rehabilitation programs, including art-based programs, being underfunded post deinstitutionalisation. They implied that psychiatric rehabilitation provision had been assigned to the non-government sector. Manager 2 elaborates:

*Then we* [*clinical mental health services*] *went generic and we all get blended in so there is really no sort of defined speciality with*[*in*] *the teams to provide that psychosocial*.

As a result, participants perceived a loss of clinical expertise in the specialty of art-based psychiatric rehabilitation within clinical services. They believed that systemic SECU factors contributed: poor unit design, minimum staffing, and a disparate resident population, with increased disabilities. The work culture was described as lacking the capacity to deliver art-based rehabilitation and recovery approaches. The absence of strengths-based practice, led to the pathologising of consumer behaviour, which the art therapist described as detrimental to staff morale and unit culture:

*I guess that staff stick together to survive that was clearly observed*, *but it becomes a culture that is very hard to shift. It is a survival mechanism so I understand why the* ‘*us versus them*’ *because it*’*s a way of surviving*.

#### Someone else’s responsibility

This theme explores the notion that existing clinical mental health services no longer have the expertise to provide rehabilitation services to consumers with enduring mental health issues, and consider this the role of external service providers. All participants agreed there was an expressed absence of a common understanding of the rehabilitation roles of government and non-government providers and art-based rehabilitation was not normally offered. Manager 2 elaborates:

*They [people diagnosed with a low prevalence disorder] will just not get a service*…*Complex personality disorder*; *we can*’*t manage them long*-*term*; *so we will pick them up to settle the crisis. But people with dual diagnosis*, *drug and alcohol*, *certainly organisations in [town name] can’t handle a lot of difficult clients. We get a lot of referrals from them*, *they have support workers*, *and they have residential programs. We try and get them back to those people*, *and then they get a bit antsy because they say they are too complex. But the reality is that we cannot hang on and we cannot manage everybody*, *we just can*’*t*.

The persistence of prejudicial language, expressed by one manager, evoked these difficult and complex issues. Participants described an area mental health service where the provision of art participation rehabilitation programs was adapted to what was accessible locally, with fewer specialised rehabilitation options the more rural the service, with responsibility assigned to external services.

#### Which service is responsible?

This theme explores the notion that clinical mental health services are confused about their role and responsibility in providing recovery-oriented rehabilitation programs especially for people with enduring mental health issues detained in the service. Most participants expressed the view that clinical services have a role in assessing consumer skills to support external rehabilitation programs and that inpatient art-based rehabilitation programs enable consumer skills and build unit relationships. Adopting a recovery-oriented rehabilitation approach in clinical services was seen as enabling clinicians to see the whole person, and provided a framework for an enabling environment. Manager 3 elaborates on the benefits for people detained in long-term care:

*We have to provide that service in*-*house*, *so that people can see that we are not just here to detain them*; *that we are here to increase their skills and interests*, *to increase their social interaction*, *and to link them in with other services*.

However, participants said the lack of guiding principles prevented the practice and provision of rehabilitation and recovery approaches across the service, including art-based programs. This was attributed to these approaches not being defined as core business, resulting in confusion concerning clinical services’ responsibility in their provision. Manager 4 explains:

*We haven*’*t actually sat down and said hang on*, *what is our main focus*? *And*, *if that should include psychosocial rehab*, *which it should*, *how do we do it*?

The consequence was that art-based recovery and rehabilitation approaches were not embedded in clinical practice, and participants expressed uncertainty about their ongoing availability in the clinical setting.

#### Where is the support?

This theme explores the notion that there is a lack of support from governing bodies and the culture of psychiatry in providing recovery-oriented rehabilitation programs for people with enduring issues detained in clinical mental health services. Participants agreed, the amount and type of funding provided for rehabilitation programs were insufficient to employ appropriately skilled staff to provide art participation rehabilitation programs to consumers living in the region. The biomedical model was identified as the dominant epistemology, which was considered a barrier to the provision of art-based recovery and rehabilitative approaches, described by manager 1:

*The resources are not often provided in terms of trained staff. The commitment is not always there from the medical perspective*, *because there is a strong medical model that drives stuff*…

#### Nevertheless, is it important

This theme explores the notion that art-based recovery-oriented rehabilitation programs are of benefit for consumers detained in the service and improve the culture and practice in clinical mental health services. All managers described benefits from art-based rehabilitation activities for consumers, staff, and the culture of inpatient units; assisting inpatients to cope with detainment and boredom, improving attitudes towards and from staff and the facility. They thought that art-based rehabilitation with qualified professionals improved a range of factors important for recovery, and the art therapist emphasised that it was the process of making art that benefitted people. The art therapist described that, in general, art therapy assisted with separating the person from the diagnosis, providing a safe environment to express emotions, recognise personal strengths, (re) discover life skills, connect with group members, and plan future goals. For consumers with an enduring mental health issue and psychosocial disability, the art therapist indicated that a renewed sense of self was possible:

*If you work long*-*term and you have several different art works from one client then they can start to recognise themselves in they*’*re style and say this is me*.

Art-based practice was seen to have broad applicability across inpatient units, reducing workloads and providing clinicians with tools to engage consumers in working collaboratively towards personal goals. The art therapist believed that if clinicians were educated to deliver and participated in therapeutic art-based group work there would be less reactive practice in clinical psychiatry:

*There would be less crisis work*, *and they won*’*t be running from one crisis to another crisis*.

## Discussion

The findings demonstrate that consumer participants found the experience of the Making Precious Things project beneficial. They identified that the program generated relational and participatory skills that enhanced their experience of the inpatient unit as a potential healing community. The process of making artwork conducted in a therapeutic environment encouraged participants to share, cooperate, and feel accepted as equals by others in the group, enabling feelings of safety and relaxation. Feeling comfortable improved participants’ self-confidence and ability to express their emotions in the group, and in their artwork, and discover personal interests and capabilities, which they did not feel was possible in the usual unit milieu. Consumers articulated many ideas to extend the rehabilitation program to support their recovery, despite complex disability and a perceived lack of service provision in this area.

While service managers and the art therapist supported the use of art-based rehabilitation programs in progressing a recovery and rehabilitation approach in clinical service provision, several systemic barriers were identified as obstructing its provision. The predominant organisational culture was characterised by a biomedical framing of recovery, understood as recovery from rather than recovery in mental illness, with responsibility for rehabilitation entrusted to external services. Barriers identified were the historic loss of clinical expertise over time in rehabilitation modalities, poor coordination between clinical and community services, and a culture deficient in recovery-oriented approaches. There was an expressed lack of understanding of recovery-oriented rehabilitation service provision, and minimal funding to support programs focussed on consumer choice, self-determination, and participation.

The literature on recovery indicates that mental health clinicians and service managers are confused by the term and meaning of what working in a recovery orientation entails [27,34], exemplified in the findings in this study. Clinicians have to balance competing epistemologies of duty of care and risk management with consumer autonomy and self-determination. Often risk management is more clearly supported by law and service policy and procedures [34]. Nurse manager participants in this study confirmed that, the predominance of a biomedical model in mental health services perpetuates negative attitudes towards those labelled mentally ill [35], exemplified by derogatory language used to describe consumers.

A consequence is the overuse of medications in clinical services with an associated decrease in a rehabilitation focus in developing therapeutic relationships with consumers [36], which encourages the repetition of the historical and cyclical condoning of a culture of coercion in mental health organisations [37]. This is despite consumer dissatisfaction with current treatments, a desire for more therapy in service provision [38] and the limited evidence of the efficacy of offering only psychotropic medications long-term [39]. Balancing different epistemologies is important for the provision of recovery-oriented care. The availability of models that can address social perspectives in mental health care is necessary [30,35]. The art therapist’s suggestion that there is less crisis work when staff uses art-based therapeutic techniques in practice supports these contentions.

Current mental health service provision discounts rehabilitation approaches because services emphasise throughput and ignore psychosocial disability, particularly in long stay clinical service populations [30]. Internationally, deinstitutionalisation has been described as under-servicing people with enduring and complex mental illness [40]. In Australia, psychiatric rehabilitation services are fragmented across clinical and community domains, with gaps in service provision prominent in rural and remote areas for people with enduring issues [41]. Despite the evidence of high levels of disability experienced by this population across financial, health, and social and relational areas [38], little clinical or community care is devoted to this area of practice [30,39]. These contentions are supported by the findings in this study. Clinical rehabilitation services are needed to assess causes and levels of psychosocial disability, and provide meaningful activities that are recovery-oriented, which include consumer-defined ambitions, and promote social inclusion [29,30]. Clinically relevant evaluation of interventions aimed at minimising the experience of psychosocial disability is essential for developing a locally informed evidence base [39].

Stigma, coercive practices, and the attitude of clinicians in inpatient services towards consumers are commonly cited issues that prevent a recovery orientation. Consequently, clinicians staffing long-stay inpatient units have little hope for people achieving recovery, and consumers regard these units as boring and dangerous with minimal staff [36]. The therapeutic relationship is conceptualised as the cornerstone of good psychiatric care [42], and important for recovery [43], but not devoid of issues related to power in psychiatric services [36]. Mental health services should be providing psychosocial interventions that aim to provide meaningful human contact, connectedness, and reciprocal relationships for this population to alleviate the profound existential suffering experienced by social isolation identified in this group [44]. The art therapist’s identification of the ‘us versus them’ culture and the ability of art-based rehabilitation programs to enhance meaningful connection in this study confirms this, and is consistent with research findings from art therapists and consumer artists [45].

Art participation programs can alleviate boredom in inpatient units [12], and assist consumers and staff to form collaborative alliances that moderate collective anxiety, fostering a healing environment [46,47]. Quantitative studies that have used methods other than RCT to determine the effects of art participation programs for people with enduring mental health issues have found improvements in mood, wellbeing, relationships, and social inclusion, particularly when art making was experienced as meaningful and engaging [48,49]. Qualitative studies consistently support the use of art making and art participation in this cohort to assist in skills development, improve psychological wellness, and promote self-expression and self-discovery [3,12,13]. The findings from this study describing consumers’ experience supports these identified benefits for consumers and staff, especially relational development and the promotion of a healing community. Research involving art therapists supports this finding, and indicates that art making is a healing tool that can promote deep human connections [25,45].

The art therapists’ emphasis on the importance of the process of art making in a safe environment in supporting recovery, is documented in literature from art therapists [25,50]. The addition of an exhibition and consumers’ description of feeling proud of the exhibited art works indicates that the product is also important, and flexibility is necessary in art-based programs to enhance both process and product outcomes [51]. The focus of the art therapist in facilitating the development of life skills, emotional expression, personal strengths, self-discovery and a renewed sense of self is generally consistent with literature from other art therapists [25,45,50], although differences exist mainly in the depth of information reported. For example, art therapists have reported that art-based practice induces a meditative state [25], whereas consumers in this study report feeling relaxed and motivated during the art making process. This difference may be because funding restraints limited the duration of sessions and the length of the program, and the focus of this study differed.

In a Korean study, psychiatrists were reported to undervalue the benefits of art therapy for people living with psychosocial disabilities [52], which may explain the findings in this study where art therapy was not usually included in inpatient services dominated by a biomedical model. For these reason, evaluation of rehabilitation programs can contribute towards developing a recovery orientation in services when consumer voices are incorporated into the range of stakeholders that inform organisational development and service provision. Reorienting resources towards evidenced-based recovery-oriented approaches needs to be based on rigorous evaluation of existing local programs. Consumer lived experience perspectives need to drive the transformation rather than continuing paternalistic clinician driven non-evidenced based programs [28]. Governance and transparency about the organisational approach is a key indicator of recovery-oriented systems [39]. These important factors are recognised and confirmed in this study.

### Limitations of the study

The results of this study are not generalisable given the small sample, qualitative design, and local area and conditions. Although data saturation was achieved, interviewing different or more participants may have changed the results. The presence of three researchers in the focus group may have influenced consumer participants’ comments towards the positive. While the art therapist’s views were important in this study, it is acknowledged that they may have had a bias towards the positive effects of art therapy. Whilst the study has limitations, description of the local experiences enhances the transferability of findings to other similar areas of practice. Using a small sample in qualitative research can provide in-depth subjective meaning to be derived [53], exemplified in the rich description of the experience of art participation and barriers to psychiatric rehabilitation in this study.

### Conclusions and implications for practice

This study is unique because it captures the experience of consumers living with enduring mental health issues detained in a SECU, a stakeholder group not well represented in literature. Many competing forces bind mental health services, and groups with the least power are often excluded from organisational decision-making. Transforming mental health services towards a recovery orientation requires commitment from service providers and their leaders, in developing evidence-based programs that are inclusive of consumer voices. Consumers, managers, and outsider perspectives can assist with understanding how art-based recovery-oriented psychiatric rehabilitation is practiced in mental health services. By understanding the tensions in a local service that prevent a recovery orientation, appropriate action and research can be undertaken to improve service provision in this important area.

This study supports the utility of recovery-oriented art-based rehabilitation programs in secure extended care facilities to ameliorate psychosocial disabilities and support a recovery orientation in clinical mental health service provision. Mental health services need to redirect funding away from an acute crisis focus to balance the services offered to people living with enduring issues. Art participation psychiatric rehabilitation programs that are based on rigorous local evaluation and consumer perspectives should be included into a recovery-oriented service. Support from organisations is necessary for these evaluations to be worthwhile [49]. Introducing art-based rehabilitation services into clinical practice with consumer perspectives prominent in service provision have the momentum to drive organisational culture change towards a recovery orientation.

## Abbreviations

GB: United Kingdom
RCT: Randomised controlled trial
SECU: Secure extended care unit
